# A Combined Prediction Model for Lymph Node Metastasis Based on a Molecular Panel and Clinicopathological Factors in Oral Squamous Cell Carcinoma

**DOI:** 10.3389/fonc.2021.660615

**Published:** 2021-04-22

**Authors:** Shu Wang, Tiancheng Li, Huan Liu, Wei Wei, Yang Yang, Chong Wang, Bo Li, Zhengxue Han, Zhien Feng

**Affiliations:** ^1^ Department of Oral and Maxillofacial-Head and Neck Oncology, Beijing Stomatological Hospital, Capital Medical University, Beijing, China; ^2^ Department of Stomatology, The Affiliated Hospital of Inner Mongolia Medical University, Hohhot, China; ^3^ Department of Otorhinolaryngology-Head and Neck Surgery, Peking University First Hospital, Beijing, China; ^4^ Clinical Epidemiology and EBM Unit, National Clinical Research Center for Digestive Diseases, Beijing Friendship Hospital, Capital Medical University, Beijing, China

**Keywords:** oral squamous cell carcinoma, gene expression profile, real-time PCR, lymph node metastasis, prediction model, CDKN2A, PLAU

## Abstract

**Objective:**

Lymph node metastasis is the most important factor influencing the prognosis of oral squamous cell carcinoma (OSCC) patients. However, there is no proper method for predicting lymph node metastasis. This study aimed to construct and validate a preoperative prediction model for lymph node metastasis and guide personalized neck management based on the gene expression profile and clinicopathological parameters of OSCC.

**Methods:**

Based on a previous study of related genes in OSCC, the mRNA expression of candidate genes was evaluated by real-time PCR in OSCC specimens. In this retrospective study, the gene expression profile and clinicopathological parameters of 112 OSCC patients were combined to construct the best prediction model for lymph node metastasis of OSCC. The model was validated with 95 OSCC samples in this study. Logistic regression analysis was used. The area under the curve (AUC) ultimately determined the diagnostic value of the prediction model.

**Results:**

The two genes CDKN2A + PLAU were closely related to lymph node metastasis of oral squamous cell carcinoma. The model with the combination of CDKN2A, PLAU, T stage and pathological grade was the best in predicting lymph node metastasis (AUC = 0.807, 95% CI: 0.713-0.881, P=0.0001). The prediction model had a specificity of 96% and sensitivity of 72.73% for stage T1 and T2 OSCC (AUC = 0.855, 95% CI: 0.697-0.949, P=0.0001).

**Conclusions:**

High expression of CDKN2A and PLAU was associated with lymph node metastasis in OSCC. The prediction model including CDKN2A, PLAU, T stage and pathological grade can be used as the best diagnostic model for lymph node metastasis in OSCC.

## Introduction

Oral cancer is a common malignant tumor that occurs in oral epithelial tissue and among these tumors, more than 90% are OSCC (1). OSCC has a propensity for occult nodal metastasis in the early stage, which is the most important factor influencing patient prognosis (2–6). Statistics have shown that the 5-year survival rate of OSCC is 50% to 60%; unfortunately, the presence of just one metastatic lymph node designates patients to an advanced stage disease category and has been shown to confer a 50% decrease in long-term survival (7). Therefore, many studies have suggested that elective neck dissection should be performed for all early-stage cN0 OSCC (2, 6). However, clinical practice clearly shows that approximately 70% of early-stage OSCC patients undergo needless neck dissections (8). To formulate individualized surgical treatment for different OSCC patients, an accurate method to judge lymph node metastasis needs to be urgently explored (9).

Many studies have found that OSCC is a polygenic disease, and gene expression profiling technology has made high-throughput gene analysis possible (10–13). Researchers can obtain the gene expression characteristics of a certain type of tumor by analyzing the gene expression profiles of tumor samples (14). Our previous research detected the expression of 22 candidate genes and 1 housekeeping gene in 120 OSCC tissue samples and 120 normal tissue samples at the mRNA level using real-time PCR (15). Statistical methods were used to analyze and determine the differentially expressed genes related to lymph node metastasis in OSCC. Cyclin-dependent kinase inhibitor 2A (CDKN2A) and urokinase-type plasminogen activator (PLAU) were closely correlated with lymph node metastasis in OSCC.

In this study, we conducted a retrospective and independent prospective large sample study of tumor tissues with tumor classification data based on the latest AJCC 8^th^ edition guidelines. The predictive value of candidate gene expression for lymph node metastasis was validated. Furthermore, the best diagnostic model for lymph node metastasis, which included CDKN2A, PLAU and other clinicopathologic parameters was analyzed.

## Materials and Methods

### Comparison of CDKN2A and PLAU mRNA Levels Between Cancerous and Normal Tissues From the Online Oncomine and GEPIA Databases

The mRNA expression data of oral cavity SCC were downloaded from the online Oncomine database (https://www.oncomine.org/). Differences in CDKN2A and PLAU expression between tumor and normal tissues were analyzed using independent sample t tests. The mRNA expression of the CDKN2A and PLAU genes in HNSCC/normal tissues was also analyzed using the online GEPIA database (http://gepia.cancer-pku.cn/).

### Prognostic Analyses of CDKN2A and PLAU Expression From the GEPIA and HUMAN PROTEIN ATLAS Databases

The association between the two genes and disease-free survival was downloaded from the online GEPIA database. The associations of CDKN2A/PLAU protein expression with 5-year overall survival for HNSCC were analyzed using the head and neck cancer - interactive survival scatter plot and survival analysis tool from the Human Protein Atlas database (https://www.proteinatlas.org/).

### Protein-Protein Interaction (PPI) Networks and Gene Set Enrichment Analysis (GSEA) of CDKN2A and PLAU From the STRING Database and GSEA Database

The PPI networks of CDKN2A and PLAU were analyzed using the STRING database (https://string-db.org/). The most primary PPI networks between the two proteins were determined. GSEA of CDKN2A and PLAU was performed using the GSEA database (https://www.gsea-msigdb.org/gsea/index.jsp).

### Patient Samples

In this retrospective study, 112 OSCC tissue specimens were selected from the Department of Oral and Maxillofacial Surgery, the Ninth People's Hospital affiliated with Shanghai Jiao Tong University School from December 2006 to March 2011.

This research was conducted in full accordance with the relevant ethical principles, including the World Medical Association Declaration of Helsinki (2002 version), and with approval of the Institutional Review Board of Beijing Stomatological Hospital (CMUSH-IRB-KJ-PJ-2020-12). Ninety-five OSCC samples were collected from Beijing Stomatological Hospital, Capital Medical University between March 2017 and January 2019. For sample selection, routine histological classification was used according to the AJCC 8^th^ edition guidelines.

In this study, the inclusion criteria for eligible patients were as follows (1): a pathological diagnosis of squamous cell carcinoma (2); a tumor located in the tongue, lower gingiva, upper gingiva, buccal mucosa, floor of the mouth, or hard palate; (3) a primary tumor without evidence of distant metastasis; (4) underwent radical resection of the primary tumor with or without neck dissection; (5) no previous treatment such as neoadjuvant chemotherapy or prior radiotherapy; (6) complete clinicopathological data, follow-up data and available tissue specimens; and (7) provided informed consent. The exclusion criteria were as follows: (1) malignancies in other organs; and (2) requested withdraw from the study.

### Real-Time PCR

After performing RNA extraction and reverse transcription on 95 fresh tissue samples of oral squamous cell carcinoma, the expression of the CDKN2A and PLAU genes was detected by real-time PCR. The β-actin housekeeping gene was used as an internal reference. All assays were carried out in triplicate. According to the protocol provided by the manufacturer, predenaturation was first performed for 30 seconds (95°C), followed by denaturation for 5 seconds(95°C) and annealing and extension for 30 seconds (60°C), for a total of 40 cycles. The primer sequences are available in **Supplemental Table 1**.

### Sample Size Calculation

The sample size calculation method was as follows: according to the conclusions of our previous retrospective study, the rate of delayed neck metastasis after OSCC surgery was approximately 50.0%, therefore, the ratio of metastatic to nonmetastatic disease was approximately 1:1. The accuracy of conventional clinical and imaging examinations in the neck for diagnosing metastasis of OSCC is approximately 60%, while a previous retrospective study found that the accuracy of predicting neck metastasis can be increased by 15% by the addition of molecular information, up to approximately 75%. Therefore, the sample size of this study was calculated as follows: applying the ratio of neck OSCC with and without actual metastasis after surgery of 1:1, PASS 15.0 software was applied for the following: One ROC Curve Power Analysis (AUC0:0.6;AUC1: 0.75), two-sided test, α=0.05 (probability of type-1 error≤5%), and β=0.20 (power of test≥80%); the minimum effective sample size to predict lymph node metastasis was 102 (51 patients in the metastatic group, and 51 patients in the nonmetastatic group). Considering the possibility of participant loss during clinical trials due to factors such as noncompliance, loss to follow-up, and accidental death, which would reduce the effective number of observed subjects, the theoretical sample size needed to be increased by 5%; thus, the sample size of this study was set to at least 108 cases.

### Statistical Analysis

**Figure 1:**
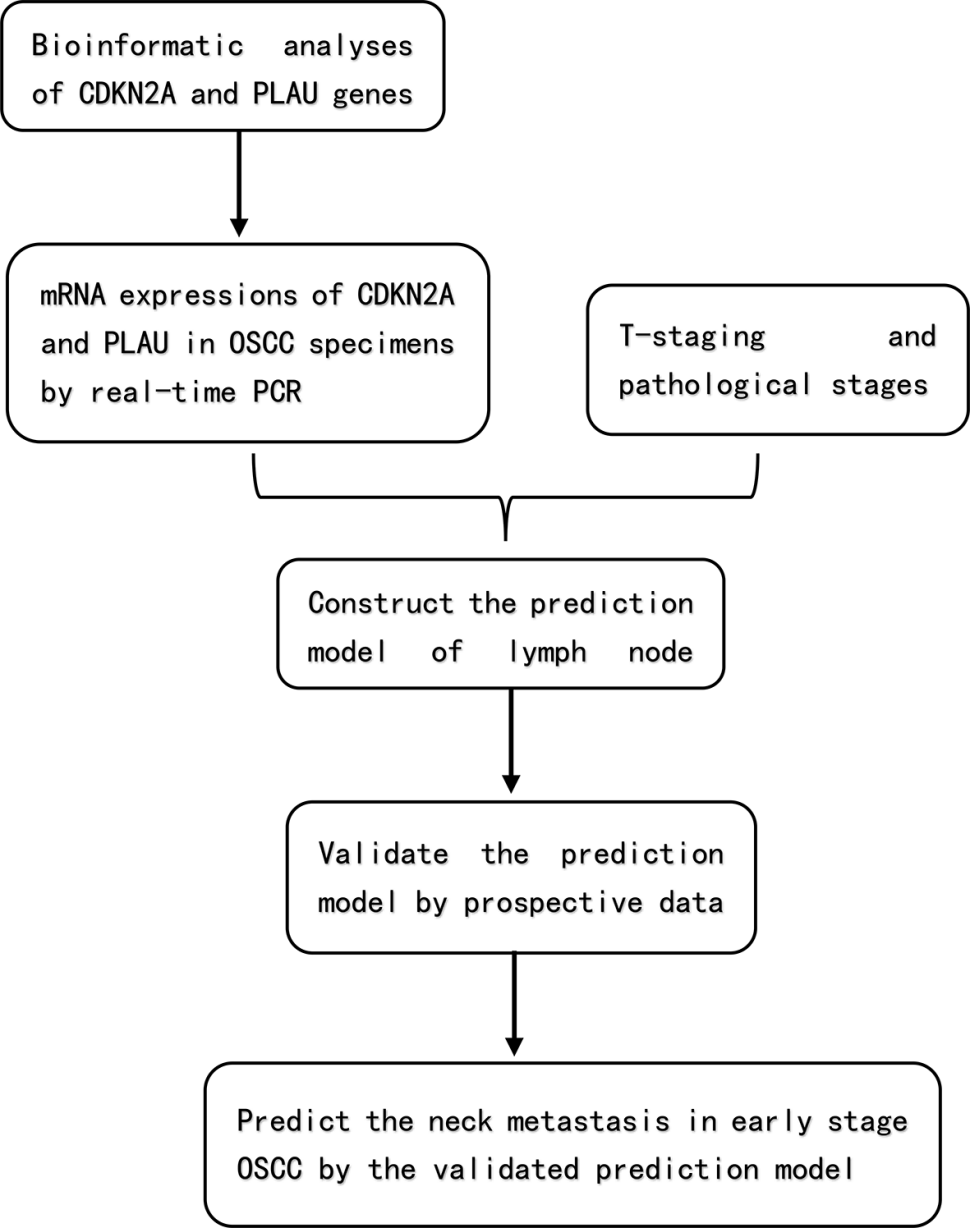
The flowchart of the study.

The flowchart of the whole study is shown in **Figure 1**. All calculations and analyses were performed using SPSS 25.0 Statistical Package for Windows (IBM Corp., Armonk, NY). The PCR data of 95 OSCC samples were standardized by the ΔCt method. The expression level was defined as 2-ΔCt, where ΔCt = Ct (target gene) -Ct (housekeeping gene). To analyze the ROC curve under logistic regression, a log transformation with base 2 was performed.

Lymph node metastasis was defined as positive cervical lymph nodes reported after neck dissection or delayed neck metastasis during the follow-up period of this study. All potential prognostic factors with P values <0.05 from the univariate analysis were incorporated into the multivariate analyses. The hazard ratios with corresponding 95% confidence intervals (CIs) and P values are reported. Logistic regression and the area under the ROC curve were used to compare and analyze the different combinations of genes and clinicopathological parameters. The ROC curve was generated by plotting the sensitivity against the false-positive rate (100-specificity), and the area under the curve (AUC) was calculated. The AUC ultimately determined the diagnostic value of the prediction model. In the case of AUCs> 0.5, the closer the AUC is to 1, the higher the diagnostic efficiency.

## Results

### Patient Characteristics

In the retrospective dataset, 75 patients were male, and 37 were female. Lymph node metastasis occurred in 71 patients with OSCC. Nineteen tumors (17.0%) were grade I, 79 tumors (70.5%) were grade II, and 14 tumors (12.5%) were grade III (**Table 1**).

**Table 1:** 112 OSCC clinicopathological parameters and gene expression analysis in retrospective data.

	metastasis group (n = 71)	nonmetastatic group (n = 41)	t/χ^2^	*P*
Age	56.82 ± 11.92	59.76 ± 10.38	1.316	0.191
Gender			0.052	0.820
male	47 (66.2%)	28 (68.3%)		
female	24 (33.8%)	13 (31.7%)		
Smoking			0.347	0.556
yes	30 (42.3%)	15 (36.6%)		
no	41 (57.7%)	26 (63.4%)		
Drinking			0.036	0.849
yes	22 (31%)	12 (29.3%)		
no	49 (69%)	29 (70.7%)		
T-staging			11.660	0.008
T1	4 (5.6%)	9 (22%)		
T2	20 (28.2%)	15 (36.6%)		
T3	19 (26.8%)	3 (7.3%)		
T4	28 (39.4%)	14 (34.1%)		
Pathological stages			8.619	0.013
I	7 (9.9%)	12 (29.3%)		
II	52 (73.2%)	27 (65.9%)		
III	12 (16.9%)	2 (4.8%)		
PLAU			4.435	0.000
CDKN2A			-1.999	0.048

In the prospective dataset, 64 patients were male, and 31 were female. The median age was 62 years (32 to 82 years). Lymph node metastasis occurred in 40 patients with OSCC, including 5 cases of extranodal extension. Ten tumors (10.5%) were grade I, 73 tumors (76.8%) were grade II, and 12 tumors (12.7%) were grade III. Regarding pathological T stage according to the AJCC 8^th^ edition guidelines, 9 patients (9.5%) were graded as T1, 27 patients (28.4%) were T2, 15 patients (15.8%) were T3, and 44 patients (46.3%) were T4 (**Table 2**).

**Table 2:** 95 OSCC clinicopathological parameters and gene expression analysis in prospective data.

	metastasis group (n = 45)	nonmetastatic group (n = 50)	t/χ^2^	*P*
Age	61.61 ± 9.25	59.30 ± 11.67	-1.077	0.284
Gender			2.790	0.095
male	26 (57.8%)	37 (74%)		
female	19 (42.2%)	13 (26%)		
Smoking			0.270	0.604
yes	24 (53.3%)	24 (48%)		
no	21 (46.7%)	26 (52%)		
Drinking			0.000	0.982
yes	17 (37.8%)	19 (38%)		
no	28 (62.2%)	31 (62%)		
T-staging (AJCC 8^th^ edition)			13.326	0.003
T1	3 (6.7%)	6 (12%)		
T2	9 (20%)	21 (42%)		
T3	9 (20%)	2 (4%)		
T4	24 (53.3%)	21 (42%)		
Depth of invasion			12.046	0.002
<5mm	5 (11.1%)	18 (36%)		
5-10mm	13 (28.9%)	18 (36%)		
>10mm	27 (60%)	14 (28%)		
Pathological stages			1.752	0.405
I	3 (6.7%)	7 (14%)		
II	35 (77.8%)	38 (76%)		
III	7 (15.5%)	5 (10%)		
CDKN2A			-2.989	0.004
PLAU			2.101	0.038

### mRNA Expression and Prognostic Value of CDKN2A and PLAU From Online Databases

**Figure 2:**
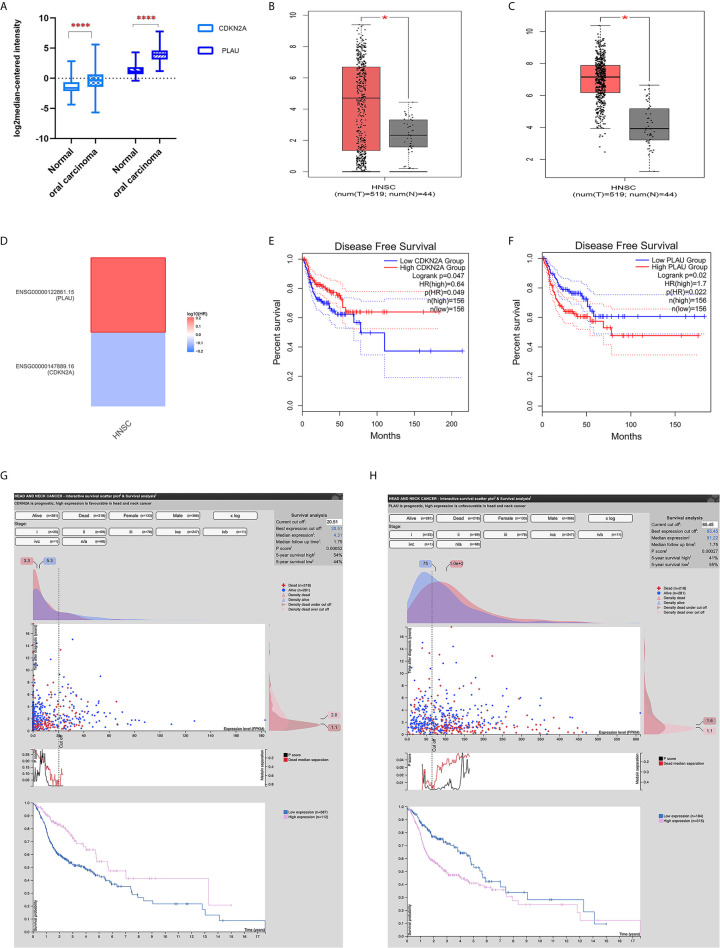
The expression of CDKN2A and PLAU and their survival significance in HNSCC from an online database. **(A)** mRNA expression of CDKN2A and PLAU in OSCC and normal tissues from the Oncomine database; **(B)** mRNA expression of CDKN2A in HNSCC from the GEPIA database; **(C)** mRNA expression of PLAU in HNSCC from the GEPIA database; **(D)** survival map displaying the prognostic significance of CDKN2A and PLAU expression in HNSCC; **(E)** CDKN2A mRNA expression and disease-free survival; **(F)** PLAU mRNA expression and disease-free survival; **(G)** interactive survival scatter plot and 5-year overall survival according to different CDKN2A protein expression; **(H)** interactive survival scatter plot and 5-year overall survival according to different PLAU protein expression. *P < 0.05; ****P < 0.01.

The expression of both the CDKN2A and PLAU genes was upregulated in cancer tissues compared with that in normal tissues in both the Oncomine database and GEPIA database. Specifically, an independent sample t test was performed on mRNA expression data of the CDKN2A and PLAU genes between OSCC (only oral cavity cancer was selected from HNSCC) and normal tissues. There were significant differences in the expression of the two genes between OSCC and normal tissues in the Oncomine database (P <0.01) (**Figure 2A**). Moreover, mRNA expression of the CDKN2A and PLAU genes in HNSCC was upregulated compared with that in normal tissues in the GEPIA database, P <0.01 (**Figures 2B, C**).

However, the results from the survival map showed that the prognostic significances of CDKN2A and PLAU expression in HNSCC were clearly different (**Figure 2D**). In the GEPIA database, high expression of CDKN2A was associated with good disease-free survival in HNSCC patients (P <0.05) (**Figure 2E**). The mRNA expression of the PLAU gene was highly expressed in HNSCC and was associated with poor disease-free survival (P < 0.05) (**Figure 2F**).

The head neck cancer - interactive survival scatter plot & survival analysis tool from the Human Protein Atlas database (https://www.proteinatlas.org/) also showed that high CDKN2A protein expression was associated with a better 5-year overall survival (high vs. low expression: 54% vs. 44%, P=0.00052, **Figure 2G**). High PLAU protein expression was associated with poor 5-year overall survival (high vs. low expression: 41% vs. 55%, P=0.00027, **Figure 2H**).

### PPI Networks Between CDKN2A and PLAU Based on the STRING Database and GSEA of CDKN2A and PLAU

According to the analysis of the STRING database, the PPI networks between CDKN2A and PLAU included cyclins, cell cycle regulation, extracellular matrix organization and the PI3K-AKT pathway (**Supplemental Figure 1**).

Analysis of the GSEA database showed, the most important respective gene sets for CDKN2A (cell cycle pathway, **Supplemental Figure 2A**) and PLAU (PI3K-AKT pathway and so on, **Supplemental Figure 2B**).

### CDKN2A and PLAU Genes Combined With Clinicopathological Parameters to Construct a Prediction Model for Lymph Node Metastasis in OSCC

#### Retrospective Data

**Figure 3:**
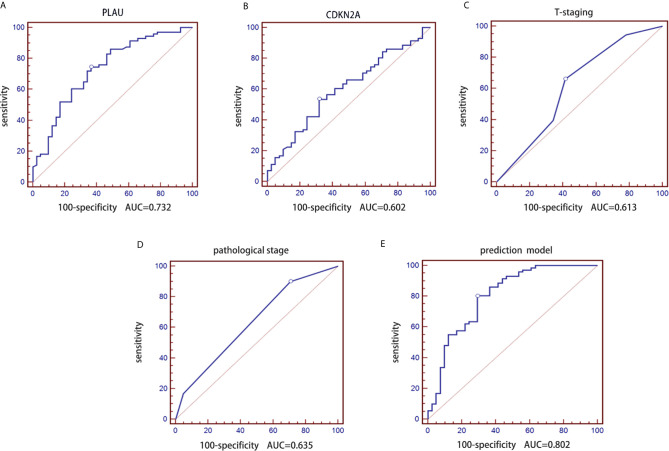
Construction of a prediction model of lymph node metastasis based on the CDKN2A and PLAU genes combined with clinicopathological parameters in the retrospective group. **(A)** ROC curve of the PLAU gene; **(B)** ROC curve of the CDKN2A gene; **(C)** ROC curve of T stages; **(D)** ROC curve of pathological grades; **(E)** ROC curve of the constructed prediction model.

First, logistic regression analysis was performed for PLAU and CDKN2A. The AUC of PLAU was 0.732 with a 95% CI of 0.640-0.811, sensitivity of 74.65% and specificity of 63.41% (**Figure 3A**). The AUC of CDKN2A was 0.602 with a 95% CI of 0.506-0.694, sensitivity of 53.52% and specificity of 68.29 (**Figure 3B**).

Second, logistic regression analysis was performed for traditional T stage and pathological grade. Similarly, the AUC of pathological T stage was 0.613 with a 95% CI of 0.516-0.704, sensitivity of 66.20 and specificity of 58.54% (**Figure 3C**). The AUC of pathological grade was 0.635 with a 95% CI of 0.539-0.724, sensitivity of 90.14% and specificity of 29.27% (**Figure 3D**).

mRNA expression data of the CDKN2A and PLAU genes in 112 OSCC samples and clinicopathological parameters (pathological T stage and grade) of the patients were used to construct a prediction model for lymph node metastasis in OSCC. We performed receiver operating characteristic (ROC) curve analysis on the real-time PCR data. We further determined the AUC and the corresponding P values from a Wilcoxon signed rank test. Additionally, we employed logistic regression analysis to identify the best combination of multiple diagnostic factors. The AUC of the final combination was 0.802 with a 95% confidence interval (CI) of 0.716-0.871, sensitivity of 80.28% and specificity of 70.73%. The corresponding ROC curve is shown in **Figure 3E**. The logistic regression equation is shown in **Table 3**.

**Table 3:** Prediction model of neck lymph node metastasis in oral squamous cell carcinoma.

	OR	95%CI	*P*	AUC	sensitivity 100%	specificity 100%
PLAU	0.063	0.012-0.324	0.001	0.732	74.65	63.41
CDKN2A	2.281	0.784-6.633	0.130	0.602	53.52	68.29
T-staging			0.139	0.613	66.20	58.54
T1	Ref					
T2	2.733	0.596-12.535	0.196			
T3	8.683	1.397-53.948	0.020			
T4	3.330	0.728-15.229	0.121			
Pathological stages			0.122	0.635	90.14	29.27
I	Ref					
II	3.038	0.874-10.563	0.080			
III	6.303	0.872-45.594	0.068			
Constant*	14.926		0.345			

Constant*; Logit(P)=2.703+0.824*(CDKN2A)-2.759*(PLAU)+1.005*T(1)+2.161*T(2)+ 1.203*T(3)+1.111* pathological stages(1)+1.841* pathological stages (2).

#### Prospective Data

**Figure 4:**
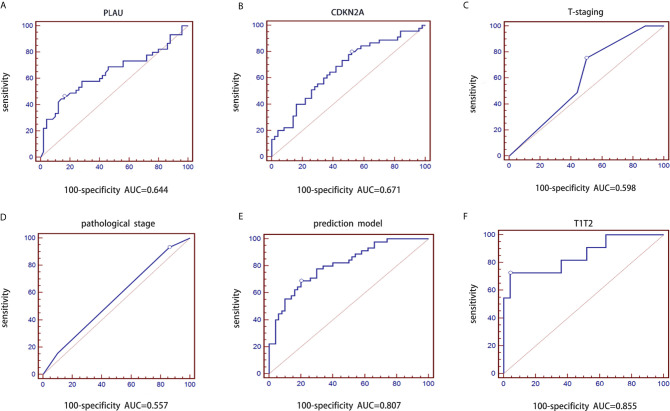
Validation of prediction model of lymph node metastasis based on the CDKN2A and PLAU genes combined with clinicopathological parameters in the prospective group. **(A)** ROC curve of PLAU; **(B)** ROC curve of CDKN2A; **(C)** ROC curve of T stages; **(D)** ROC curve of pathological grades; **(E)** ROC curve of validated prediction model for the whole group; **(F)** ROC curve of validated prediction model for T1/T2 stage subgroup.

We conducted a prospective large sample study of 95 OSCC patients combined with tumor pathological T stage classified according the latest AJCC 8th edition guidelines. The model constructed from the retrospective dataset was used to predict the probability of lymph node metastasis in the independent validation dataset. Similarly, logistic regression analysis was performed based on PLAU, CDKN2A, T stage and pathological grade. The purpose of using the ROC curve is to evaluate the difference in diagnostic efficacy (sensitivity and specificity) between the predictive model and the gold standard of clinical diagnosis. The AUC of PLAU was 0.644 with a 95% CI of 0.539-0.739, sensitivity of 46.67% and specificity of 84.00% (**Figure 4A**). The AUC of CDKN2A was 0.671 with a 95% CI of 0.567-0.764, sensitivity of 80.00% and specificity of 48.00% (**Figure 4B**). The AUC of pathologic T stage was 0.598 with a 95% CI of 0.493-0.698, sensitivity of 75.56% and specificity of 50.00% (**Figure 4C**). The AUC of pathological grade was 0.557 with a 95% CI of 0.451-0.659, sensitivity of 93.33% and specificity of 14.00% (**Figure 4D**). The AUC for the prediction model including CDKN2A and PLAU mRNA expression, T stage and pathological grade of OSCC was 0.807 with a 95% CI 0.713-0.881, sensitivity of 68.89% and specificity of 80.00% (**Figure 4E**). The logistic regression equation is shown in **Table 4**.

**Table 4:** Value analysis of prediction model in prospective data.

	AUC	sensitivity (100%)	specificity (100%)
PLAU	0.644	46.67	84.00
CDKN2A	0.671	80.00	48.00
T-staging	0.598	75.56	50.00
Pathological stages	0.557	93.33	14.00
PLAU+CDKN2A+ T-staging + pathological stages	0.807	68.89	80.00

### The Combination of CDKN2A and PLAU mRNA Expression, T Stage and Pathological Grade Forms the Best Prediction Model for Lymph Node Metastasis in Early-Stage OSCC

The diagnostic performance of the prediction model for early stage OSCC was further evaluated. The same model was applied to early-stage samples(T1/T2). The corresponding AUC for patients with T1 and T2 stage disease was 0.855 with a 95% CI of 0.697-0.949 (sensitivity: 72.73% and specificity: 96.00%, **Figure 4F**). This result indicated that the diagnostic performance of the prediction model was optimal for early-stage OSCC patients.

## Discussion

The main feature of OSCC metastasis is that it easily spreads along draining lymphatics, and it is relatively uncommon for OSCC to metastasize to distant sites (16). However, cN0 OSCC as an indication for elective neck dissection in all patients is still controversial. Traditional T staging and pathological grading increasingly show the limitations of predicting metastasis. Many novel pathological factors have been considered as potential approaches to predict the risk of regional recurrence, in particular the number of positive lymph nodes and lymph node ratio (17–20). However, to obtain the results of the above two variables, it is necessary that the patient has undergone neck dissection and can only judge the risk of postoperative neck recurrence. It does not apply to the prospective weighing of whether the patient should undergo neck dissection or neck observation strategy. Accordingly, the influence of biological heterogeneity on OSCC metastasis is increasingly recognized (21). In recent years, many biomarkers have been developed to predict lymph node metastasis, but there is no one or panel of markers in the field of OSCC that can be widely applied in clinical practice (22). Trying to integrate biomarkers and clinicopathological variables and predict jointly is a more feasible strategy (23, 24).

With the update of the AJCC 8^th^ edition guidelines in 2017, the novel T stage, which fully considers the increased value of depth of invasion for the clinical staging of early tumors, was believed to improve predictive discrimination over that of the AJCC 7^th^ edition guidelines (25, 26). Pathology grade was an important part of routine pathology reports despite its controversial prognostic value (27). Our recent study found that pathological grade had independent prognostic value in early-stage OSCC but not in advanced-stage OSCC (28). In the prospective dataset, the T stage (AJCC 8^th^ edition guidelines) and pathological grade were included in the predictive model as the crucial traditional variables. Although the two variables partially reflect a predictive value for lymph node metastasis, neither of the two variables alone nor in combination can achieve very good discrimination. Therefore, the inclusion of highly effective biomarkers in this model will be a key factor in establishing a metastasis prediction model.

As expounded by Yalniz et al. (12), the metastasis of OSCC can be predicted, and multiple accurate prediction profiles can be obtained by using various predictive gene subsets. Our previous study explored a molecular diagnostic method based on real-time quantitative PCR technology to determine lymph node metastasis in OSCC. CDKN2A and PLAU were identified as closely related genes to metastasis of OSCC. The mRNA expression of the CDKN2A and PLAU genes in OSCC tumor tissues was higher than that in normal tissues. The CDKN2A gene encodes the tumor suppressor protein p16, which prevents phosphorylation of retinoblastoma protein and thus halts the cell cycle progression from G1 to S phase (29). Downregulated expression, inactivation or copy number deletion of CDKN2A has been a frequent event in the development of OSCC and is related to the occurrence, development and prognosis of OSCC (29–33). In addition, it was also found that a CDKN2A/p16 (+) status in head and neck cancer was strongly predictive of poorly differentiated tumors (34). The above findings showed that CDKN2A was associated with an increased clinical stage and histological differentiation of OSCC. PLAU belonging to the S1 serine peptidase of Clan PA is a proteinase involved in the transformation of plasminogen to plasmin, and it can hydrolyze extracellular matrix remodeling related proteins and activate growth factors (35). Extracellular matrix organization and the P13K-Akt signaling pathway may be involved in the possible mechanism of PLAU’s function in OSCC (35). PLAU and its receptor were upregulated in tumor cells and were associated with tumor proliferation, migration and metastasis (36–40).

A recently published study based on the Gene Expression Omnibus (GEO) and TCGA databases identified and validated a set of robust prognostic signatures including PLAU, CLDN8 and CDKN2A, that could predict overall survival in OSCC patients (41). Gene ontology (GO) enrichment analysis, ingenuity pathway analysis (IPA), PPI network and survival analysis indicated that their three-gene signature and identified several pathways that play important roles in regulating the initiation and development of OSCC. As there were few genes that overlapped with the findings of different gene expression profiling studies with similar purposes (42), this key study also indirectly indicated that our predictive markers can be replicated between different studies. What differentiates out study from previous research is that our study combined biomarkers with clinicopathological characteristics and used tissue samples to confirm that we collected this model has a good ability to predict lymph node metastasis in early OSCC. The direct or indirect PPI networks between CDKN2A and PLAU include cyclins, cell cycle regulation, extracellular matrix organization and the PI3K-AKT pathway, which regulate proliferation, invasion and metastasis.

In this prospective study, T stage was defined according to the latest AJCC 8^th^ edition guidelines. The AUC for the prediction model was 0.807 with a sensitivity of 68.89% and specificity of 80.00%. The specificity of the predictive model for lymph node metastasis in OSCC tumor tissues increased by nearly 10% compared with that in the retrospective study. Furthermore, the specificity of the model was increased to 96% for T1 and T2 stage OSCC tumor tissues. In other words, the true-negative rate of the prediction model was 96%; thus, these low-risk patients did not need to undergo neck dissection. This avoids wasting health-care resources and improves quality of life.

The present study included a retrospective training set and a prospective validation set. The main limitation of the study was that the T stages were classified according to the AJCC 7^th^ edition classification in the training set. DOI and extranodal extension data are also partially missing. However, the research was a two-center study, and in both independent samples of OSCC, the model achieved high predictive efficiency for lymph node metastasis, especially in early-stage diseases. Thus, the study has good external authenticity. These limitations will be given further consideration in future studies.

## Conclusions

Our study demonstrates that this prediction model has considerable clinical value for the accurate diagnosis of lymph node metastasis of OSCC. Before the model is applied in clinical practice, a randomized controlled trial is still needed.

## Data Availability Statement

The original contributions presented in the study are included in the article/**Supplementary Material**. Further inquiries can be directed to the corresponding author.

## Ethics Statement

The studies involving human participants were reviewed and approved by the Institutional Review Board of Beijing Stomatological Hospital (CMUSH-IRB-KJ-PJ-2020-12). The patients/participants provided their written informed consent to participate in this study.

## Author Contributions

SW: project administration, validation, writing-original draft, interpretation of data. TL: data analysis, writing-original draft, interpretation of data. HL: validation, writing-original draft, interpretation of data. WW: design of the work, interpretation of data, revising it critically for the intellectual content. YY, CW, BL: resources, writing-original draft, interpretation of data. ZH: supervision, writing-review & editing, resources. ZF: funding acquisition, project administration, supervision, writing-original draft, interpretation of data. All authors contributed to the article and approved the final version.

## Funding

This work was supported by the Beijing Municipal Administration of Hospitals Clinical Medicine Development of Special Funding Support (XMLX201819); the Capital’s Funds for Health Improvement and Research (CFH2020-2-2143); the National Natural Science Foundation of China (82072984); the Program of Beijing Municipal Education commission (KM202110025008); and the Shanghai Science and Technology Committee (18DZ2291500).

## Conflict of Interest

The authors declare that the research was conducted in the absence of any commercial or financial relationships that could be construed as a potential conflict of interest.

## Supplementary Material

Supplementary Table 1 Primer sequences

Supplementary Figure 1 PPI networks between CDKN2A and PLAU based on the STRING database

Supplementary Figure 2 GSEA of CDKN2A and PLAU based on the GSEA database
