# Supplementary material for: A Combined Prediction Model for Lymph Node Metastasis Based on a Molecular Panel and Clinicopathological Factors in Oral Squamous Cell Carcinoma
Source: Front Oncol. 2021 Apr 22;11:660615. doi: 10.3389/fonc.2021.660615 (PMC8100439; doi:10.3389/fonc.2021.660615)
Supplement: Supplementary Table 1 — Primer sequences [file DataSheet_1.zip › supplemental materials/Supplemental Table1.docx]

Supplemental table 1. primer sequences

| gene primer sequences (5'--3') |
| --- |
| F-PLAU GTCGTGAGCGACTCCAAAGGCA  R-PLAU TTCACAGTGCTGCCCTCCGAA  F-CDKN2A CCGCCGCGAGTGAGGGTTTT  R-CDKN2A CGCTGCCCATCATCATGACCTGG  F-actin CCTGGCACCCAGCACAAT  R-actin GGGCCGGACTCGTCATACT |
