# Supplementary figures and images for: A Combined Prediction Model for Lymph Node Metastasis Based on a Molecular Panel and Clinicopathological Factors in Oral Squamous Cell Carcinoma
Source: Front Oncol. 2021 Apr 22;11:660615. doi: 10.3389/fonc.2021.660615 (PMC8100439; doi:10.3389/fonc.2021.660615)

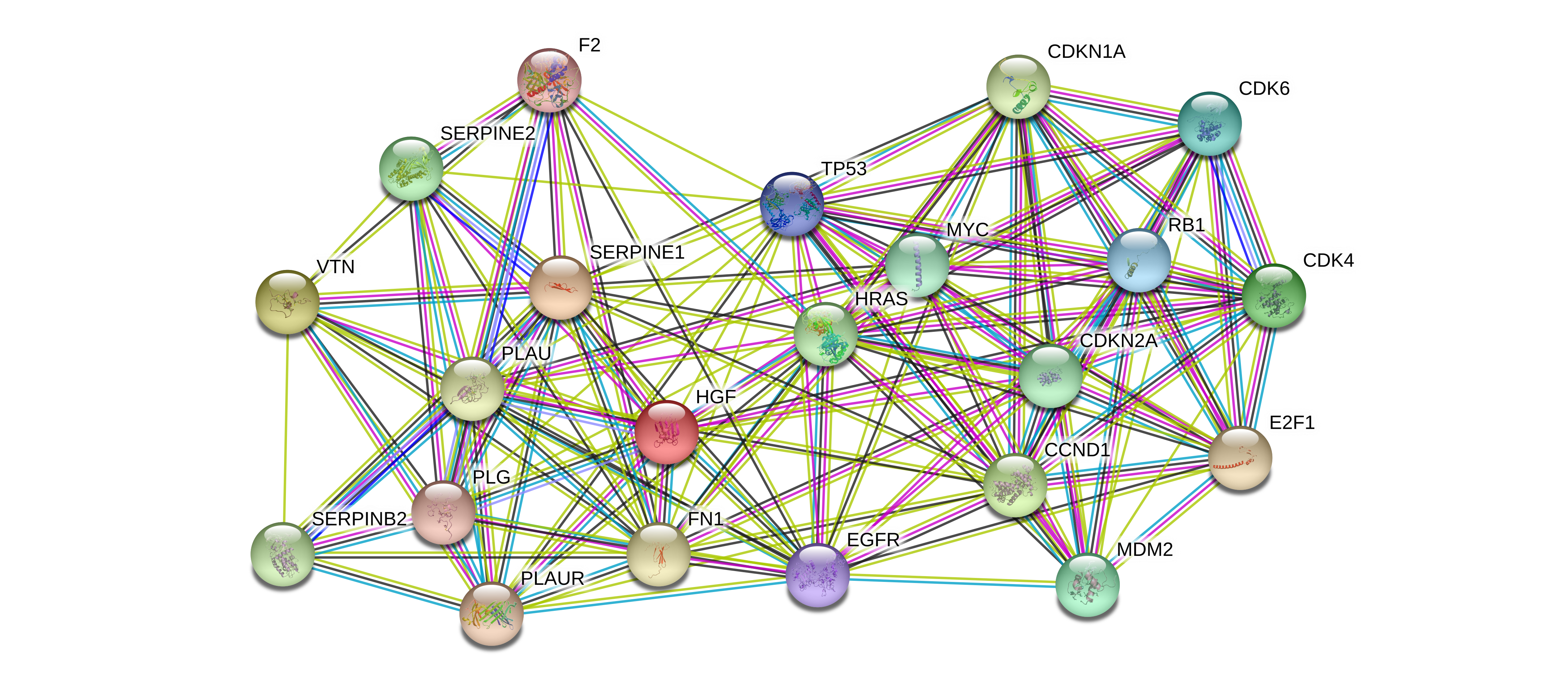

Supplement: Supplementary Table 1 — Primer sequences [file DataSheet_1.zip › supplemental materials/Supplemental Figure 1.png]

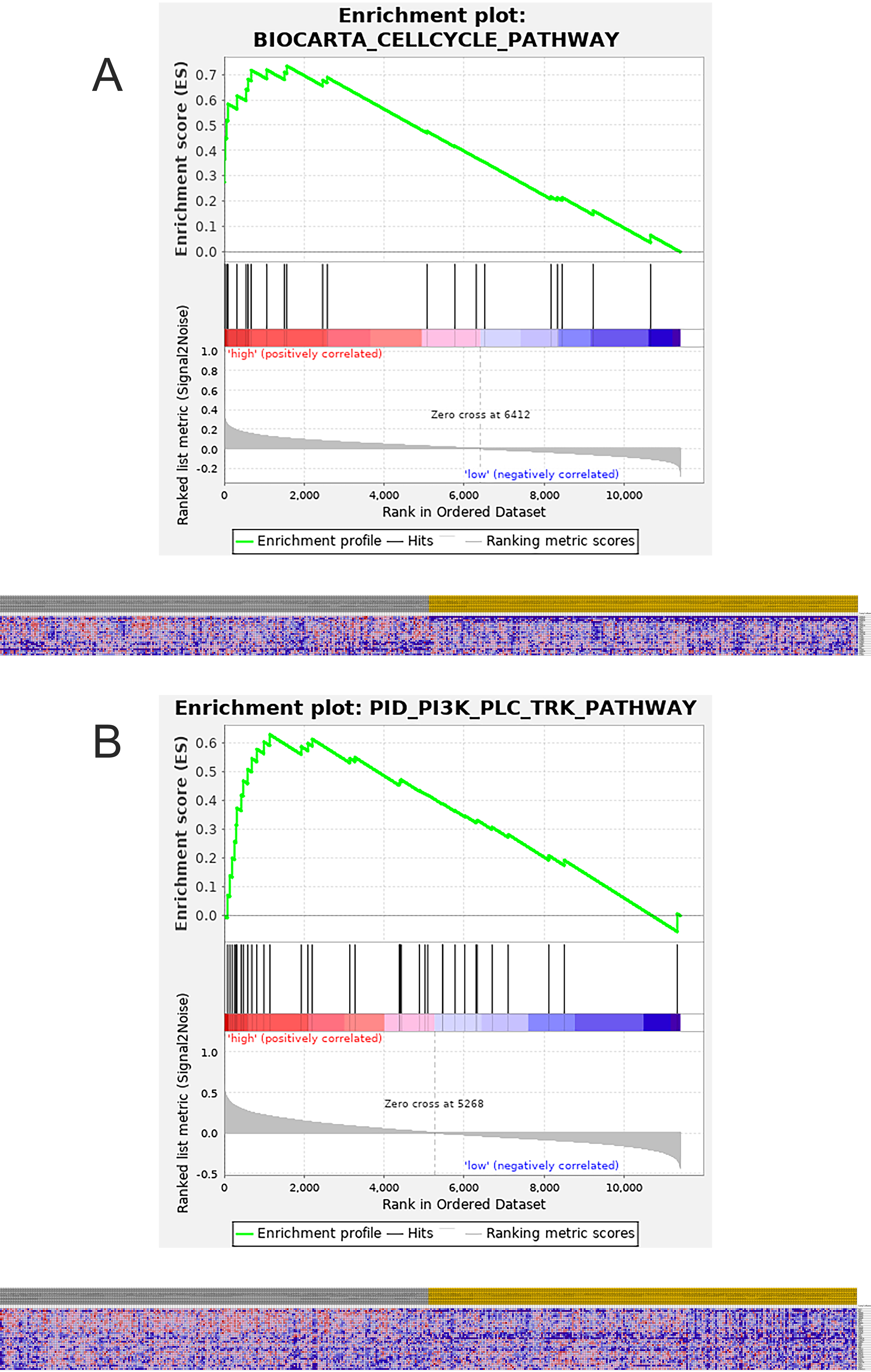

Supplement: Supplementary Table 1 — Primer sequences [file DataSheet_1.zip › supplemental materials/Supplemental Figure 2.tif]
